# B Cell IgD Deletion Prevents Alveolar Bone Loss Following Murine Oral Infection

**DOI:** 10.1155/2009/864359

**Published:** 2009-10-25

**Authors:** Pamela J. Baker, Nicole Ryan Boutaugh, Michaela Tiffany, Derry C. Roopenian

**Affiliations:** ^1^Biology Department, Bates College, Lewiston, ME 04240, USA; ^2^Immunology/Inflammation/Hematology Section, The Jackson Laboratory, Bar Harbor, ME 04609, USA

## Abstract

Periodontal disease is one of the most common infectious diseases of humans. Immune responses to infection trigger loss of alveolar bone from the jaw and eventual tooth loss. We investigated the contribution of B cell IgD to alveolar bone loss by comparing the response of B cell normal BALB/cJ mice and IgD deficient BALB/c-Igh-5^−/−J^ mice to oral infection with *Porphyromonas gingivalis*, a gram-negative periodontopathic bacterium from humans. *P. gingivalis*-infected normal mice lost bone. Specific antibody to *P. gingivalis* was lower and oral colonization was higher in IgD deficient mice; yet bone loss was completely absent. Infection increased the proportion of CD69^+^ activated B cells and CD4^+^ T cells in immune normal mice compared to IgD deficient mice. These data suggest that IgD is an important mediator of alveolar bone resorption, possibly through antigen-specific coactivation of B cells and CD4^+^ T cells.

## 1. Introduction

Periodontal diseases are infectious diseases of humans and other mammals that result in damage to the soft tissue and periodontal ligaments of the gingiva, resorption of alveolar bone from the jaw, and, ultimately, loss of teeth. It is becoming increasingly apparent that there is extensive interaction and cross-talk between the immune system and bone remodeling. This new concept has wide-ranging implications for how we think about the causes and regulation of bone resorption and has engendered a new field called osteoimmunology [1, 2]. Bone is constantly remodeling, with osteoblasts building bone and osteoclasts breaking it down. In a state of health, these two processes are in balance and the density of bone remains stable. This balance is disrupted in various diseases, including the osteoclastic resorptive disease, chronic periodontitis. In this periodontal disease the progressive loss of alveolar bone is largely activated by immune responses to a small subset of the more than 600 species of bacteria that colonize the oral cavity [1]. In a murine model that we have developed previously, we have shown that alveolar bone loss from the jaw can be triggered by oral infection with one of these 600 oral bacterial species, *Porphyromonas gingivalis*, a periodontopathic gram-negative anaerobe from humans [3, 4]. We, and others, have shown that in response to oral infection B lymphocytes and CD4^+^ T lymphocytes of the immune system trigger bone resorption [1, 3–5].

Despite the prevalence of periodontal disease, the pathways to bone loss are not fully understood. In the most well-characterized pathways, infection upregulates expression of ligand for receptor activator of NF-*κ*B (RANK-L) on specific antigen-activated B and CD4^+^ T lymphocytes. These immune cells are then able to bind to RANK on preosteoclasts, triggering bone resorption [4–7]. We wished to explore whether there are other immune cell surface molecules that can induce bone loss. In this study we have used mice deficient in their B lymphocyte expression of IgD, a B cell surface immunoglobulin known to be involved in B cell/T cell cognate activation, but not known to be involved in bone remodeling.

In immune normal mice and humans, expression of membrane bound IgD along with IgM indicates that a B cell has undergone affinity maturation and can begin to react with specific antigen and secrete soluble antibody to that antigen. Igh-5^−/−^ mice have a targeted mutation that deletes expression of heavy chain-5 (Igh-5), the delta-like heavy chain of IgD immunoglobulin [8]. Mice deficient in IgD display a delay in B cell affinity maturation [8]. In addition, B cell membrane IgD is a ligand for IgD receptors that are expressed on activated CD4^+^ T cells [9]. Binding of IgD to IgD receptors facilitates B cell antigen presentation to CD4^+^ T cells by stabilizing T cell receptor binding to the antigen/MHC complex. This cognate interaction increases both antibody responses by the B cells and antigen-specific clonal expansion of the T cells [9]. We therefore designed experiments using Igh-5^−/−^ mice to test the hypothesis that IgD can contribute to alveolar bone loss in response to *P. gingivalis* infection.

## 2. Materials and Methods

### 2.1. Animals

Specific pathogen-free BALB/c-Igh-5^−/−J^ mice that are IgD deficient due to a specific deletion of the *Igh* gene [8] and the genetically matched immune normal strain BALB/cJ were received from The Jackson Laboratory (Bar Harbor, ME, USA). Mice were kept in the animal colony at Bates College under conditions described previously [10]. The experimental protocol was reviewed and approved by the Institutional Animal Care and Use Committee, Bates College.

### 2.2. Bacteria

*P. gingivalis* ATCC 53977 (A7A1-28) was maintained frozen in defibrinated sheep's blood at −80°C. For experiments, bacteria were anaerobically grown under 5% CO_2_, 10% H_2_ and 85% N_2_ at 37°C for 4–7 days on supplemented blood agar containing trypticase soy agar base with 0.1% yeast extract, hemin (5.0 *μ*g/mL), menadione (0.5 *μ*g/mL), and 5% defibrinated sheep's blood. Bacteria were removed from the plates, gently suspended in phosphate-buffered saline (PBS), and diluted to 10^10^ colony forming units (CFU) per mL on the basis of optical density. Pilot experiments in which serial dilutions of *P. gingivalis* were plated for colony counts had shown that a 1:100 dilution of 10^10^ CFU per mL of *P. gingivalis* has an optical density of 0.3 at 650 nm.

### 2.3. Oral Infection

Each experiment included ten age-matched female mice, 14 to 16 weeks old at the start of experiments. Mice were given sulfamethoxazole (0.87 mg/mL) and trimethoprim (0.17 mg/mL) (Sulfatrim, Goldline Laboratories, Ft. Lauderdale, FL, USA), ad libitum in their drinking water for 10 days, followed by a 3-day antibiotic-free period. Mice were then infected by gavage needle and direct oral exposure three times at 2-day intervals with 10^9^ CFU of live *P. gingivalis* in 100 *μ*L of PBS with 2% carboxymethylcellulose. Controls received the antibiotic pretreatment and three sham-infections with carboxymethylcellulose without *P. gingivalis*. Forty-two days after the final gavage, mice were euthanized by CO_2_ [3]. Duplicate experiments yielded the same results; the results from one experiment are shown.

### 2.4. Measurement of Alveolar Bone Loss

Horizontal bone loss around the maxillary molars of defleshed skulls was assessed under a dissecting microscope (×40) fitted with a video image marker measurement system (model VIA 170, Boeckeler Instruments, Inc., Tucson, AZ, USA) standardized to give measurements in fractions of mm. The distance from the cementoenamel junction (CEJ), where the enamel of the tooth crown meets the cementum of the tooth root, to the alveolar bone crest of the jaw (ABC) was measured at a total of 14 buccal sites per mouse (for location of measurement sites see photographs in [11]). Measurements were done in a blinded protocol by one investigator.

As alveolar bone resorbs away, additional root cementum is exposed and the distance from the CEJ to the new location of the ABC becomes larger. Where the CEJ to ABC is larger in infected mice than in sham-infected mice, bone loss has occurred. The CEJ to ABC distance is not zero; consequently, the CEJ to ABC distance at individual sites in infected mice was subtracted from the mean of the distance at that site in sham-infected mice, giving the mm of change in bone at that site.

### 2.5. Reisolation of *P. gingivalis* from the Oral Cavity

At the time of euthanasia, bacterial samples were collected from the oral cavity using one sterile paper-point per mouse placed along the CEJ for 5 seconds on the buccal surface of the three maxillary molars on the left and 5 seconds along the molars on the right. Each sample was individually plated and incubated as described previously [10].

### 2.6. *P. gingivalis*-Specific Antibody

Blood was collected from each mouse at the time of euthanasia. Sera were stored at −80°C for later assessment of specific IgM, IgG, and IgA antibody by enzyme-linked immunosorbent assay (ELISA), as described previously [10], in polystyrene plates (Costar, Corning Inc., Corning, NY, USA) coated with formalin-killed whole *P. gingivalis* ATCC 53977. The ELISA titer was defined as the reciprocal of the highest serum dilution (expressed in log_2_) that produced absorbance readings more than two standard deviations above background levels. The *P. gingivalis*-specific titer of each infected animal was calculated by subtraction of the mean value from the sham-infected animals of that mouse strain.

### 2.7. Flow Cytometry

Spleens were collected at the time of euthanasia. Fifty *μ*L of mouse spleen cells at a density of 2 × 10^7^ cells/ml were washed in 4 ml of wash buffer (0.2 g KCl, 8.0 g NaCl, 1.15 g Na_2_HPO_4_, 0.2 g KH_2_PO_4_ and 0.2 g NaN_3_ per liter at pH 7.2) and pelleted at 110 × g for 10 minutes at 15°C. The pellets were resuspended in 1 ml of the same wash buffer and were blocked with 10 *μ*L of normal rat IgG (Sigma Chemical Company, St. Louis, MO, USA) and incubated for 15 minutes at 7°C. Cells were immunostained for 30 minutes at 7°C with either 10 ul of prediluted hamster antimouse CD69 conjugated to FITC to show activation status, or phycoerythrin-conjugated hamster antimouse CD19 to mark B cells or hamster anti-mouse CD4 to mark CD4^+^ T cells, and FITC- or phycoerythrin-labeled hamster IgG as isotype controls (The Jackson Laboratory, Bar Harbor, ME, USA). Unadsorbed antibody was removed by adding wash buffer and centrifuging at 60 × g for 10 minutes at 10°C. Cells were resuspended in 0.5 ml flow cytometry buffer (the wash buffer with 5 ml/l fetal bovine serum added). Five *μ*L of propidium iodide at 20 *μ*g/ml were added to determine cell viability. Data were acquired on a FACSORT (Becton-Dickinson, San Jose, CA, USA) and were analyzed using CellQuest. Lymphocytes were gated on the basis of forward scatter and side scatter of incident light [12].

### 2.8. Statistics

Differences between the infected and sham groups and between infected mice of different strains were evaluated by two-tailed, unpaired *t*-tests (Excel; Microsoft, Redmond, WA, USA).

## 3. Results

**Figure 1:**
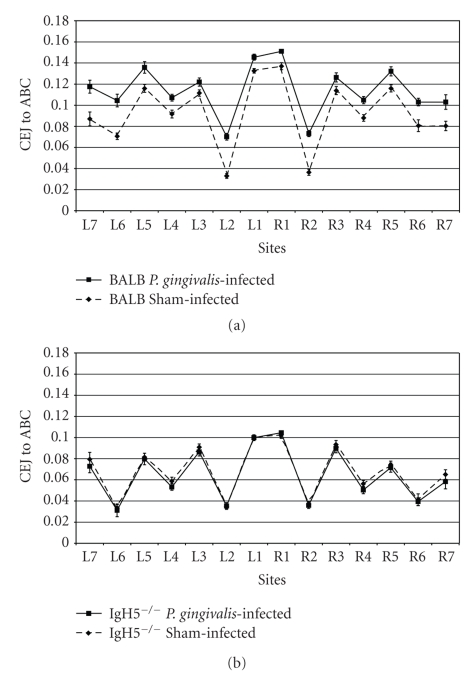
Changes in bone levels due to oral infection with *P. gingivalis*. The distance from the CEJ on the tooth to the ABC of the jaw was measured at 14 buccal sites on the maxillary molars, seven sites on the left (L) and seven on the right (R), with site number 1 located closest to the midline. Each point indicates the mean measurement at that site ±1 SEM (*n* = 10 mice). (a) Immune normal BALB/cJ mice showed alveolar bone resorption when infected with *P. gingivalis*. The distances from the CEJ to the ABC were significantly larger in infected mice than in sham-infected mice (*P* < .05) at each of the 14 sites, indicating that alveolar bone resorbed at every measurement site. (b) IgD deficient IgH5^−/−^ mice did not show alveolar bone resorption. The distances from the CEJ to the ABC were the same in *P. gingivalis*-infected mice as in sham-infected mice (*P* > .05).

The effects of oral infection with *P. gingivalis* on alveolar bone are shown in Figure 1. In BALB/cJ mice with normal IgD levels the CEJ to ABC distance was larger in infected mice than in sham-infected mice at each of the 14 measurement sites, indicating that the infected BALB/cJ mice had lost bone at every site (Figure 1(a)). In contrast, in IgD deficient mice the CEJ to ABC distances were the same in infected mice as in sham-infected mice, showing that without B cell IgD mice did not lose bone at any site (Figure 1(b)).

Summing the amount of bone change at all 14 sites additionally demonstrated that bone loss occurred in immune normal mice but not in IgD deficient mice. The summed change in infected B cell normal BALB/cJ mice was a mean of −0.302 ± 0.035 mm of bone. In contrast, the summed bone change in infected IgD deficient mice was 0.040 ± 0.024 mm (*P* = 1.28 × 10^−7^).

To be sure that lack of bone loss could not be explained by a lack of infection, we reisolated *P. gingivalis* from the oral cavities by paper point. *P. gingivalis* was present in each of the infected mice of both strains and was not present in any of the uninfected mice (data not shown). The *P. gingivalis* load was actually much higher (a mean of 100 ± 10 colonies per plate, *n* = 10) in the IgD deficient mice than in the immune normal mice where the mean was only 2 ± 1 colonies per plate (data not shown).

**Figure 2:**
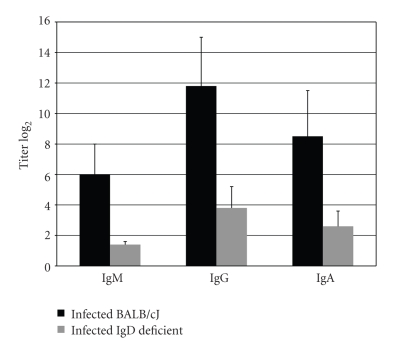
*P. gingivalis-*specific serum antibody titers of all three isotypes were significantly lower in infected IgD deficient mice compared with immune normal BALB/cJ mice (*P* < .05). Bars represent the mean anti-*P. gingivalis* titer of infected mice ± SEM (*n* = 10) after subtraction of the mean value for sham-infected mice of the same strain.

The difference in bacterial load may reflect differences between the mouse strains in their production of *P. gingivalis*-specific serum antibody. After infection, both mouse strains produced a specific antibody response significantly higher (*P* < .05) than the background levels in sham-infected animals (data not shown); however, the titers of each of the three antibody isotypes in the infected mice were lower in the IgD deficient mice than in the immune normal mice (*P* < .05) (Figure 2).

**Figure 3:**
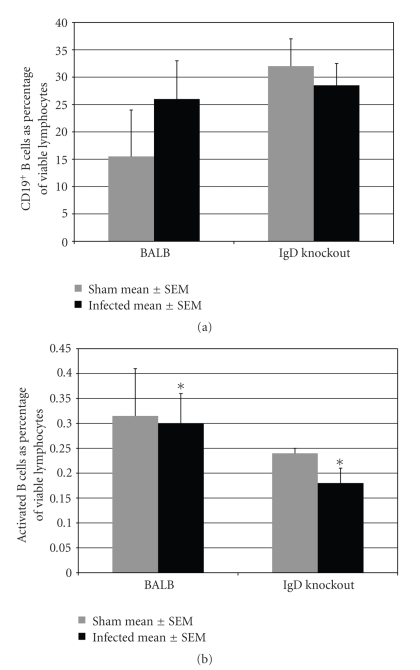
*P. gingivalis-*infected IgD deficient mice had a lower proportion of activated B cells than infected immune normal BALB/cJ mice*.* (a) The total B cell population: the percentage of total viable CD19^+^ B cells in the sham-infected mice were the same in both strains (*P* > .05). In neither mouse strain was the total B cell population changed by *P. gingivalis* infection (*P* > .05, infected compared to sham-infected mice). (b) Activated B cell population: the proportion of activated B cells in infected IgD deficient mice were significantly lower than in infected BALB/cJ mice, (*P* < .05, indicated by *) *n* = 8; 42 days postinfection.

Despite the differences in serum antibody titers, Igh-5^−/−^ mice are known to have normal proportions of B cells [8]. This was confirmed in our experiments by flow cytometry measurements on the day of euthanasia, 42 days postinfection by comparing the sham-infected mice of the two strains. The mean percentage of B cells was higher in IgD knockout mice than in normal mice, but the difference between the two mouse strains was not statistically significant (Figure 3(a)). In neither mouse strain did *P. gingivalis*-infection change the percentage of total B cells compared with sham-infected mice (Figure 3(a)). Although total B cell percentages were unchanged, a higher percentage of B cells were activated in infected BALB/cJ mice than in infected IgD knockout mice (*P* < .05; Figure 3(b)).

**Figure 4:**
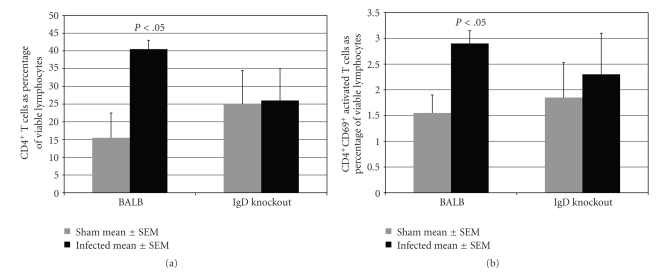
*P. gingivalis-*infection increased the proportion of CD4^+^ T cells and activated them in immune normal BALB/cJ mice but not in IgD deficient mice*.* (a) Total CD4^+^ T cell population: in immune normal BALB/cJ mice the percentage of total viable CD4^+^ T cells were higher in infected than in sham-infected (*P* < .05) but in IgD deficient mice the percentage was unchanged by infection (*P* > .05). (b) Activated CD4^+^ T cells: In BALB/cJ mice the proportion of activated CD4^+^ CD69^+^ T cells were higher in *P. gingivalis*-infected than in sham-infected mice (*P* < .05) but activation was not increased by infection in IgD deficient mice (*P* > .05). *n* = 10.

We also examined the CD4^+^ T cell populations in the two mouse strains. In BALB/cJ mice *P. gingivalis*-infection led to a significant increase in the mean percentage of total viable CD4^+^ T cells compared with sham-infected BALB/cJ mice (*P* < .05). In IgD deficient mice, however, infection did not increase the CD4^+^ T cell population (Figure 4(a)). Similarly, infection activated CD4^+^ T cells in BALB/cJ mice, as shown by the increased percentage of CD69^+^ CD4^+^ T cells, but it did not activate T cells in IgD deficient mice (Figure 4(b)).

## 4. Discussion

In this study, we show that mice with B cells lacking surface IgD do not experience alveolar bone loss after oral infection with *P. gingivalis* (Figure 1). One possible explanation for the lack of bone loss could be that the mice were colonized by with a diminished load of *P. gingivalis*. Reisolation of the bacteria eliminated this possibility; IgD deficient mice actually harbored higher numbers of this bacterium than immune normal mice. Higher colonization may be due to the lower antibody titers found in the IgD deficient (Figure 2). These reduced titers are in line with earlier studies showing that diminished antibody response is due to the delayed affinity maturation characteristic of this IgD mutation [8]. Previous studies in immune normal mice have demonstrated that antibody may decrease bacterial load but antibody is not protective against bone loss: lower antibody titers do not lead to more bone loss [13].

We cannot entirely exclude another possibility; that is, that the resistance to bone loss seen in the IgD deficient mice was due to residual contamination with alleles from the 129 or C57BL/6 progenitor strains. The *Igh* knockout was originally generated on the C57BL/6 genetic background by gene targeting with an embryonic stem cell line derived from murine strain 129 [8]. BALB/c-Igh-5^−/−J^ mice were produced by backcross onto the BALB/cJ strain for seven generations. Strain BALB/cJ is genetically susceptible to bone loss after oral infection with *P. gingivalis*, while strains 129 and C57BL/6 show moderate resistance to bone loss in some experiments [11]; however, mice within each cohort do lose bone after infection. In seven backcross generations the progenitor genotypes are predicted to be diluted 1 in 2^7^, leaving less than 0.8% of the progenitor alleles carried into the BALB/cJ genome and then only if they are closely linked to the target *Igh* gene. In the experiments presented in this paper, no infected BALB/c-Igh-5^−/−J^ mouse showed an increase in the CEJ to ABC distance (our measure of bone loss) that was beyond one standard error of the mean value of the shams; that is, the distance from the CEJ to the ABC in every infected mouse was within the range of variation of the sham mice. We therefore think it highly unlikely that the absence of bone loss observed in the infected mice is the result of passenger 129 or C57BL/6 gene loci but is instead an intrinsic feature of the *Igh* deficiency.

We propose that the resistance to bone loss is due to the inability of IgD deficient B cells to act as costimulators of antigen-specific T cells. T lymphocytes and other cells are normal in Igh-5^−/−^ mice [8]. T cells have IgD receptors that are lectins and bind to O-glycans on B cell surface IgD immunoglobulin [14]. These IgD receptors are present on very low numbers of peripheral T cells but are moved to the surface membrane in response to activation by antigen [9]. The upregulated IgD receptor on an antigen-specific T cell binds to IgD on a B cell bearing the same antigen, stabilizing the T cell receptor/MHC II connection between the two types of lymphocytes, facilitating activation of both.

Other forms of costimulation have been shown to be important in alveolar bone loss. Inflammatory bone resorption triggered by *Aggregatibacter* (formerly *Actinobacillus*)* actinomycetemcomitans* requires activation of Th1 CD4^+^ T cells, and this activation requires costimulation of B7 on the T cell surface. Blocking of B7 abrogated costimulation and inhibited bone resorption in a rat model [15]. The present results suggest that B cell IgD binding to CD4^+^ T cell IgD receptors is a pathway to costimulation of both B cells and CD4^+^ T cells in *P. gingivalis*-induced disease. Our experiments show that oral infection of immune normal mice with *P. gingivalis* leads to both serum antibody response (Figure 2, an indication of B cell activation) and to higher numbers and activation of CD4^+^ T cells (Figure 4). In contrast, Igh-5^−/−^ mice lacking IgD showed signs of less activation of both B and T cells. They produced lower antibody titers (Figure 2), had fewer activated B cells (Figure 3), and did not show an increase in T cell population or activation at 42 days after infection (Figure 4).

The mechanism by which this cognate B and T cell activation stimulates bone loss is not known at this time. It is not likely to be due to the effect of IgD deletion on antibody secretion, as antibody is not protective against bone loss [13]. One well-studied immunological pathway to bone loss is upregulation of RANK-L on activated lymphocytes. Both B cells and T cells express membrane bound and soluble RANK-L after activation [16, 17], and bone loss can be triggered by the binding of this ligand to RANK on preosteoclasts. However, both CD4^+^ and CD8^+^ T cells can express RANK-L after activation [16]. We have shown that CD4^+^ T cells and their cytokines are involved in alveolar bone loss in mice, but that CD8^+^ T cells are not [3]. Others have also implicated CD4^+^ cells [2, 15, 18], suggesting that there may a pathway in addition to RANK-L that is particular to CD4^+^ but not CD8^+^ T cells. Although we have some preliminary data showing that the proportion of CD4^+^ spleen cells expressing membrane surface RANK-L was the same in infected immune normal mice that lost bone as it was in IgD deficient mice that did not lose bone (data not shown), we have not examined RANK-L expression on T or B cells in the gingiva.

One pathway that could distinguish between CD4^+^ and CD8^+^ T cells is the pathway suggested by these experiments, that is, activation through the T cell IgD receptor. In mice, only CD4^+^ T cells express IgD receptors and so CD8^+^ T cells cannot participate in the costimulation [9]. CD4^+^ T cell/ B cell interaction may provide sufficient amplification of RANK-L upregulation that the cells triggering osteoclasts are largely restricted to B cells and CD4^+^ T cells. Han et al. have shown that adoptive transfer of antigen-responsive B cells can induce bone loss in rats that are congenitally athymic and therefore T cell deficient [19]. It is possible that B cells are alone responsible for the effects on bone loss seen in these experiments, but the activation of CD4^+^ T cells over an extended period of time (42 days postinfection; Figure 4) would argue that T cell activation is playing at least an amplifying role, either through RANK-L or through increased secretion of bone resorptive cytokines [3, 20].

## 5. Conclusion

Genetic deletion of IgD was shown here to diminish T and B cell activation and to inhibit alveolar bone loss in mice. This study suggests a new pathway through which oral infection with *P. gingivalis* can result in loss of alveolar bone. In response to oral infection, IgD on the surface of B cells binds to IgD receptors on CD4^+^ T cells, resulting in coactivation of both B and T cells of the immune system. Such B and T cell activation can, in turn, activate osteoclasts and trigger bone loss. Chronic periodontitis is a widespread infectious disease; therefore, elucidation of the mechanisms through which the immune responses to infection trigger damage rather than protection is of clinical importance.
